# Polypharmacy and multiple sclerosis: A population-based study

**DOI:** 10.1177/13524585221122207

**Published:** 2022-10-27

**Authors:** Anibal Chertcoff, Huah Shin Ng, Feng Zhu, Yinshan Zhao, Helen Tremlett

**Affiliations:** Faculty of Medicine (Neurology), University of British Columbia and The Djavad Mowafaghian Centre for Brain Health, UBC Hospital, Vancouver, BC, Canada; Faculty of Medicine (Neurology), University of British Columbia and The Djavad Mowafaghian Centre for Brain Health, UBC Hospital, Vancouver, BC, Canada/College of Medicine and Public Health, Flinders University, Bedford Park, SA, Australia; Faculty of Medicine (Neurology), University of British Columbia and The Djavad Mowafaghian Centre for Brain Health, UBC Hospital, Vancouver, BC, Canada; Faculty of Medicine (Neurology), University of British Columbia and The Djavad Mowafaghian Centre for Brain Health, UBC Hospital, Vancouver, BC, Canada; Faculty of Medicine (Neurology), University of British Columbia and The Djavad Mowafaghian Centre for Brain Health, UBC Hospital, Vancouver, BC, Canada

**Keywords:** MS, polypharmacy, population-based data, pharmacoepidemiology, prescription medication use

## Abstract

**Background:**

Little is known about polypharmacy and multiple sclerosis (MS).

**Objectives:**

To estimate polypharmacy prevalence in a population-based MS cohort and compare persons with/without polypharmacy.

**Methods:**

Using administrative and pharmacy data from Canada, we estimated polypharmacy prevalence (⩾5 concurrent medications for >30 consecutive days) in MS individuals in 2017. We compared the characteristics of persons with/without polypharmacy and described the number of polypharmacy days, the most common medication classes contributing to polypharmacy and hyper-polypharmacy prevalence (⩾10 medications).

**Results:**

Of 14,227 included individuals (75% women), mean age = 55.4 (standard deviation (SD): 13.2) years; 28% (*n* = 3995) met criteria for polypharmacy (median polypharmacy days = 273 (interquartile range (IQR): 120–345)). Odds of polypharmacy were higher for women (adjusted odds ratio (aOR) = 1.14; 95% confidence intervals (CI):1.04–1.25), older individuals (aORs 50–64 years = 2.04; 95% CI:1.84–2.26; ⩾65 years = 3.26; 95% CI: 2.92–3.63 vs. <50 years), those with more comorbidities (e.g. ⩾3 vs. none, aOR = 6.03; 95% CI: 5.05–7.22) and lower socioeconomic status (SES) (e.g. most (SES-Q1) vs. least deprived (SES-Q5) aOR = 1.64; 95% CI: 1.44–1.86). Medication classes most commonly contributing to polypharmacy were as follows: antidepressants (66% of polypharmacy days), antiepileptics (47%), and peptic ulcer drugs (41%). Antidepressants were most frequently co-prescribed with antiepileptics (34% of polypharmacy days) and peptic ulcer drugs (27%). Five percent of persons (716/14,227) experienced hyper-polypharmacy.

**Conclusion:**

More than one in four MS persons met criteria for polypharmacy. The odds of polypharmacy were higher for women, older persons, and those with more comorbidities, but lower SES.

## Introduction

Polypharmacy, defined as the concurrent use of multiple medications, has been associated with a higher risk of adverse drug reactions and drug interactions leading to negative health outcomes and an increased risk of hospitalization and visits to the emergency department.^1^ Polypharmacy has been identified by the World Health Organization (WHO)^2^ as a major and growing public health concern globally, and across healthcare settings. In North America, around 10%–15% of adults more than 40 years and 30%–35% of those older than 65 years reported taking at least five medications concurrently (2015–2017).^3^

People with MS may be at higher risk of accruing multiple medications (polypharmacy) for several reasons: MS can lead to symptoms compromising quality of life that may require management with different drug therapies;^4^ accumulation of comorbidities may warrant medication treatment(s);^5^ and adverse effects associated with the disease-modifying drugs (DMDs) used to treat MS may require co-administration of other medications.^6^

Although the concurrent use of multiple drugs can be clinically appropriate, there is also evidence to suggest that once started, medications can be difficult to discontinue. This can lead to their accumulation over time, even though the original indication may no longer be relevant, or has long been forgotten.^7^ To date, most studies assessing medication use in MS, beyond just the DMDs, have been either fairly small,^8,9^ not population based,^10,11^ or have focused mainly on drug costs.^12^

The aim of our study was to estimate the prevalence of polypharmacy over 1 entire calendar year, in a large, population-based cohort of people with MS, and to examine the characteristics of those with and without polypharmacy.

## Methods

### Data sources and population

We accessed health administrative and pharmacy data in the universal healthcare setting of British Columbia (BC), Canada. The datasets were linked (using encrypted unique personal healthcare numbers for each individual) and de-identified before analyses, and consisted of the Medical Services Plan Payment Information File (capturing physician visit dates and diagnoses, coded using the International Classification of Diseases (ICD-9) system); the Discharge Abstract Database (capturing the dates of, and reasons for, hospitalizations, coded using the ICD-9/10 system); Vital Statistics (capturing death dates); the Medical Services Plan Registration and Premium Billing Files (providing demographics, socioeconomic and residency status); and PharmaNet (providing records of all prescriptions filled at community/outpatient pharmacies, irrespective of the payer. Prescription medications costs in BC are covered from a variety of sources, including publicly funded programs offered by federal and provincial governments (typically based on a person’s age, income, or medical condition), employment-linked drug coverage plans, and direct out-of-pocket payments.^13^ PharmaNet does not capture over-the-counter medications or products purchasable without a prescription (e.g. supplements). SES estimates, expressed as quintiles, were obtained via Statistics Canada’s software which links each individual’s postal code to census-derived neighborhood income.^14^

We identified MS cases via a validated algorithm requiring individuals to have ⩾3 ICD diagnostic codes specific for MS (ICD-9/10 340/G35) in the physician and hospital data, or a prescription for ⩾1 MS-specific DMD in the pharmacy data (Supplemental Table).^15^ The study entry date was 1 January 2017, and the main observation period was the 2017 calendar year. Individuals ⩾18 years with their first MS-specific or demyelinating event (Supplemental Table) captured before 1 January 2017 were potentially eligible. Included individuals also had to be resident in BC for ⩾90% of the days in the observation year (2017) and the year prior to ensure adequate capture of the outcome (polypharmacy) and cohort characteristics. The cohort characteristics, assessed at study entry, included sex, age, SES quintiles (Q1–Q5), and comorbidity burden (categorized as 0, 1, 2, and ⩾3), measured using the Charlson Comorbidity Index, based on hospital and physician ICD codes in the year pre-study entry (excluding hemiplegia/paraplegia to avoid misclassifying MS as a comorbidity).^16^

### Outcomes

**Figure 1.**
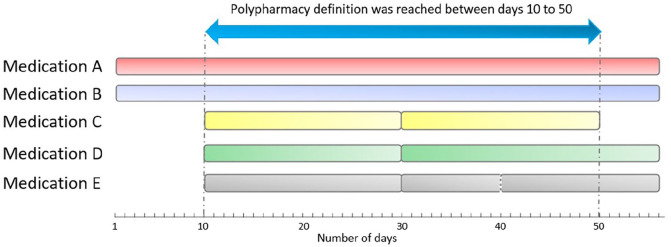
Illustrative example of an individual meeting the definition of polypharmacy: concurrent exposure to at least five different medications for more than 30 consecutive days. *Medications A-E, represent five individual, unique medications (WHO’s ATC classification system, fifth level). The definition of polypharmacy was met as concurrent exposure to ⩾5 medications occurred between Day 10 and Day 50. The total number of polypharmacy days was 41. Using Medication E as an example to illustrate how overlapping prescription fills for the same drug were handled: on Day 40, another prescription was filled (dotted vertical line) before the previous supply of this medication was potentially exhausted. Thus, the last day of exposure for this medication was estimated based on the days of drug supplied at Day 40 (i.e. at the last prescription fill).

The main outcome was the prevalence of polypharmacy in 2017, defined as the concurrent exposure to ⩾5 medications for >30 days consecutively (illustrated in Figure 1).^17^ Medication exposure was determined by using the prescription fill date and days of drug supplied. If overlapping fills for the same drug were recorded, the days supply from the last fill was used to estimate the end of exposure for that drug (Figure 1). To account for possible early fills pre-study entry, the outcome was estimated using prescriptions filled from 1 October 2016 to 31 December 2017.^18^ All prescriptions were mapped to the WHO Anatomical Therapeutic Chemical (ATC) classification system’s fifth level (“chemical substance,” i.e. unique drug, such as metformin) and third level (“medication classes”) to obtain an overview of the most frequently prescribed classes and their combinations.

### Statistical analysis

Polypharmacy prevalence and cohort characteristics were described. The association of the cohort characteristics (sex, age (<50 years, 50–64 years, and ⩾65 years), SES quintiles, and comorbidity burden categories with polypharmacy status (exposed/unexposed) was examined using logistic regression, with findings expressed as aORs with 95% CIs.

For individuals with polypharmacy, we also described: The total number of polypharmacy days summed for each person (grouped: 31–180, 181–270, and 271–365 days); the number of individual medications and medication classes per person (ATC fifth level and third level, each grouped as 5, 6–7, 8–9, and ⩾10); the most commonly prescribed medication classes, and the most common combination of medication classes contributing to polypharmacy. The prevalence of hyper-polypharmacy (⩾10 individual medications concurrently for >30 consecutive days) was also described, primarily because of its emerging interest as an extreme form of polypharmacy.^19^

As complementary approaches, we: i) described the proportion of DMD users/non-users in 2017, their characteristics (at study entry) and prevalence of polypharmacy; and ii) examined the association between polypharmacy status and all-cause hospitalizations (any vs. none) during 2017 using sex, age, SES quintiles, and comorbidity burden (grouped: 0, 1, 2, and ⩾3) adjusted logistic regression.

Analyses were conducted using *R* (v4.1.0; R Foundation for Statistical Computing, Vienna, Austria).

## Results

### Cohort characteristics and polypharmacy prevalence

**Figure 2.**
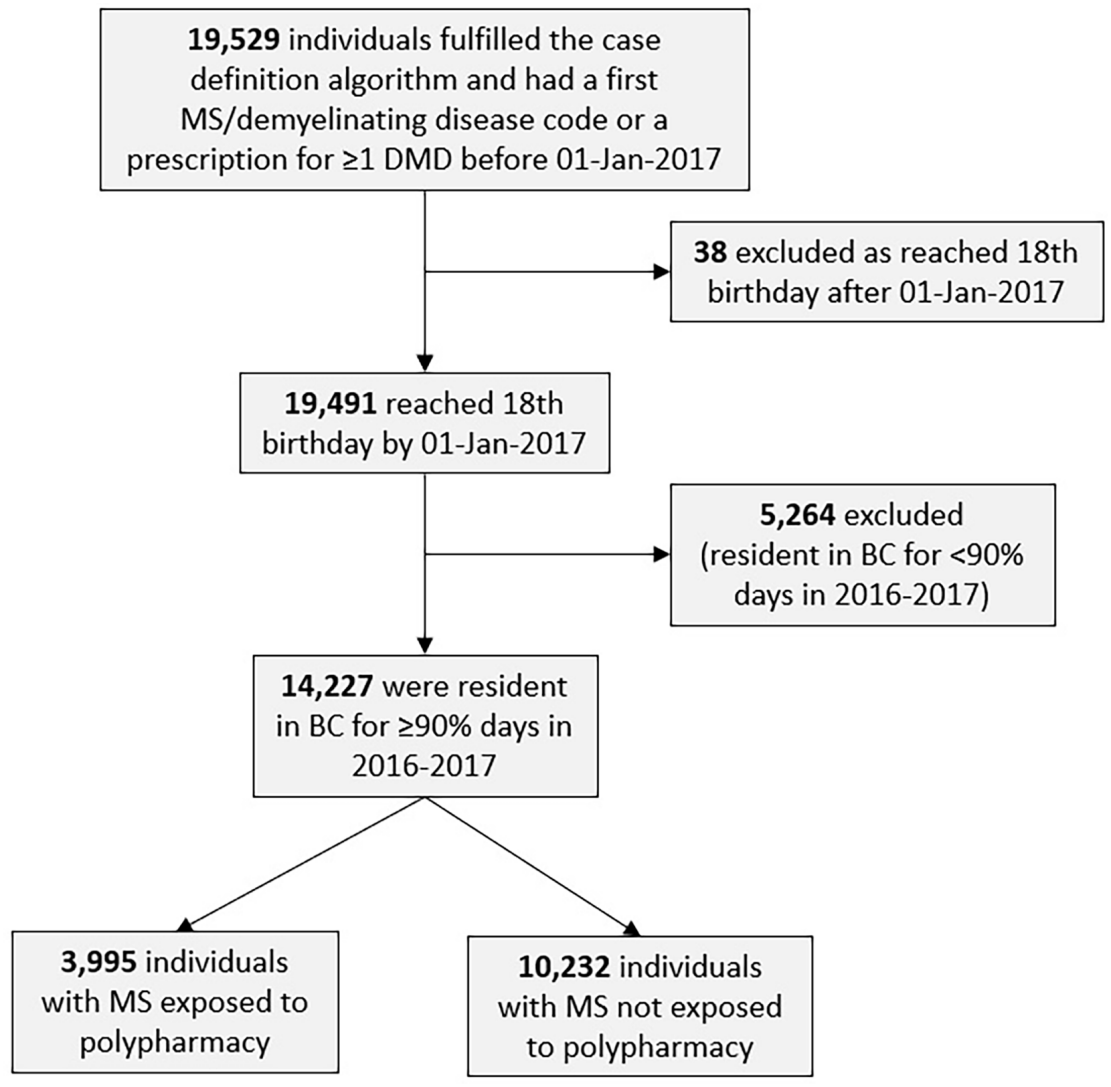
Flowchart of individuals with MS included in the study. MS: Multiple sclerosis; DMD: disease-modifying drug; BC: British Columbia.

The study cohort included 14,227 individuals with MS (Figure 2, Table 1), and 74.5% were women. At study entry, the mean age was 55.4 years (SD: 13.2) and 4160 individuals (29.2%) had ⩾1 comorbid condition measured by the Charlson Comorbidity Index. The prevalence of polypharmacy in the MS population was 28.1% (3995/14,227) during the study year (2017).

**Table 1.** Characteristics at study entry of the MS population with and without polypharmacy over the study period (year 2017).

	Exposed to polypharmacy (⩾5 concurrent medications for >30 days), *n* = 3995	Not exposed to polypharmacy, *n* = 10,232
**Sex**, *n* (%) Women	3007 (75.3)	7590 (74.2)
**Age in years**, mean (SD)	60.8 (11.9)	53.3 (13.1)
**Age group**, *n* (%)		
<50 years	717 (17.9)	3990 (39.0)
50 to 64 years	1738 (43.5)	4257 (41.6)
⩾65 years	1540 (38.5)	1985 (19.4)
**SES**, *n* (%)^a^		
1 (most deprived)	878 (22.0)	1742 (17.0)
2	885 (22.2)	1886 (18.4)
3 or missing^b^	799 (20.0)	2101 (20.5)
4	760 (19.0)	2309 (22.6)
5 (most affluent)	673 (16.8)	2194 (21.4)
**Comorbidity score**, *n* (%)^c^		
0	1955 (48.9)	8112 (79.3)
1	1143 (28.6)	1350 (13.2)
2	491 (12.3)	559 (5.5)
⩾3	406 (10.2)	211 (2.1)

SD: Standard deviation. ^a^SES quintiles were derived from each individual’s postal code linked to their neighborhood-level income; ^b^Information was missing for 17 (0.4%) of the polypharmacy-exposed subjects and for 52 (0.5%) of the unexposed. ^c^Comorbidity score was measured by using the Charlson Comorbidity Index (excluding hemiplegia/paraplegia) during the year before study entry. This instrument was used to evaluate the population overall comorbidity burden.

**Figure 3.**
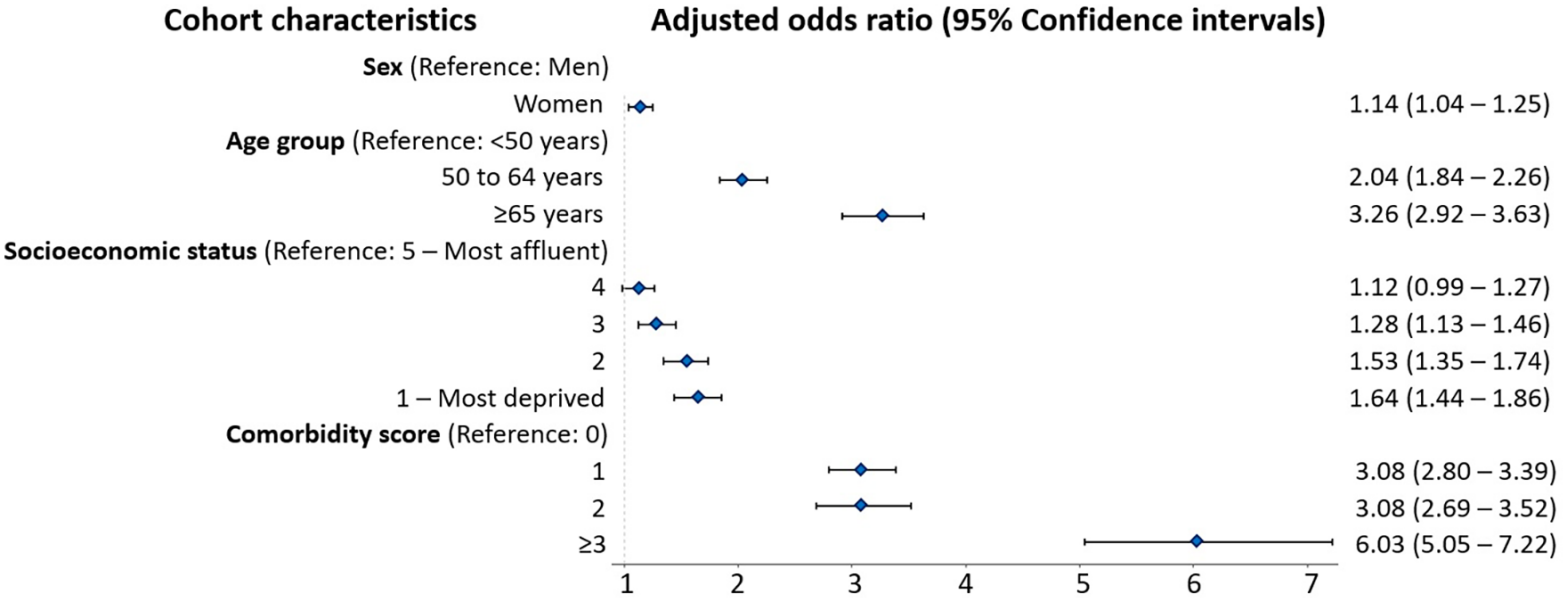
Association between the characteristics of the MS population at study entry and the presence of polypharmacy (⩾5 concurrent medications for >30 days) in 2017. Cl: Confidence interval. Results were obtained from a logistic regression model. aORs represent the odds of polypharmacy accounting for the other characteristics shown in the Figure at study entry. SES quintiles were derived from each individual’s postal code linked to their neighborhood-level income. Comorbidity score was measured by using the Charlson Comorbidity Index (excluding hemiplegia/paraplegia) during the year before study entry.

The odds of polypharmacy (Figure 3) were higher for women than men (aOR = 1.14; 95% CI: 1.04–1.25), older individuals (aORs 50–64-year-olds = 2.04; 95% CI: 1.84–2.26; ⩾65-year-olds = 3.26; 95% CI: 2.92–3.63 relative to younger individuals, <50-years-old), and for those with more comorbidities (e.g. ⩾3 relative to no comorbidities aOR = 6.03; 95% CI: 5.05–7.22) and lower SES (e.g. most (SES-Q1) vs. least deprived (SES-Q5) aOR = 1.64; 95% CI: 1.44–1.86).

### Polypharmacy days, number of medications, and hyper-polypharmacy

For those with polypharmacy, the total number of polypharmacy days of exposure was 935,364 (median per individual: 273; IQR: 120–345), and 64.6% of persons were exposed for longer than 180 days (Table 2).

**Table 2.** Polypharmacy days, number of individual medications, and number of medication classes among persons with MS exposed to polypharmacy in 2017.

	Number of polypharmacy days, total = 935,364	Number of individuals exposed to polypharmacy, *n* = 3995
**Polypharmacy days**, *n* (%)		
31–180	130,279 (13.9)	1417 (35.5)
181–270	127,293 (13.6)	562 (14.1)
271–365	677,792 (72.4)	2016 (50.5)
**Number of individual medications (ATC fifth level)**, *n* (%)*		
5	276,103 (29.5)	369 (9.2)
6–7	349,553 (37.4)	1412 (35.3)
8–9	173,179 (18.5)	1075 (26.9)
⩾ 10	136,529 (14.6)	1139 (28.5)
**Number of medication classes (ATC third level)**, *n* (%)*		
⩽5	372,983 (39.9)	642 (16.1)
6–7	324,725 (34.7)	1475 (36.9)
8–9	148,757 (15.9)	994 (24.9)
⩾10	88,899 (9.5)	884 (22.1)

ATC: Anatomical Therapeutic Chemical classification system (from the WHO). *Each individual’s contribution to the “Number of polypharmacy days” varied according to the number of medications (ATC fifth level)/medication classes (ATC third level) prescribed on any given day. The “Number of individuals exposed to polypharmacy” was based on the maximum number of concurrent individual medications/medication classes at any one time during the study period.

At any one time, more than half (55.4%) of persons with polypharmacy were exposed to eight or more individual medications (and 47.0% to eight or more medication classes). Exposure to ⩾8 individual medications accounted for 33.1% of the total polypharmacy days (and 25.4% of the days for the medication classes).

In addition, 5.0% (716/14,227) of the whole MS population had evidence of hyper-polypharmacy (⩾10 concurrent individual medications for >30 days).

### Most frequent medication classes/medication combinations

The medication classes most commonly contributing to polypharmacy, based on the number of polypharmacy days, were the antidepressants (66.2% of polypharmacy days), the antiepileptics (47.3%, with gabapentin, clonazepam, and pregabalin as the most frequent), the drugs for peptic ulcer and gastro-esophageal reflux disease (GERD) (41.0%), the lipid-modifying agents (33.0%), and the centrally acting muscle relaxants (27.2%) (Table 3). In addition, among polypharmacy-exposed individuals, 43.3% (1730/3995) filled a prescription for an opioid versus 14.9% (1528/10,232) of those polypharmacy-unexposed.

**Table 3.** Most frequent prescriptions filled by persons with MS exposed to polypharmacy in 2017, shown as medication classes ranked based on the number of polypharmacy days, and specifying their potential to elicit central nervous system (CNS) effects per class.

Ranking (high to low by polypharmacy days)	Medication class (ATC third level)	Polypharmacy days, % (*n* = 935,364) ‡	Individuals, % (*n* = 3995) ‡	CNS effects †
1	Antidepressants	66.2	67.9	++
2	Antiepileptics	47.3	51.6	++
3	Drugs for peptic ulcer/GERD	41.0	45.4	−/+
4	Lipid-modifying agents	33.0	32.0	−
5	Centrally acting muscle relaxants	27.2	33.9	++
6	ACE inhibitors	25.0	25.6	−
7	Opioids	23.5	43.3	++
8	Thyroid preparations	22.5	22.1	−
9	Beta blocking agents	20.2	19.8	−/+
10	Hypnotics and sedatives	18.4	24.9	++
11	Antithrombotic agents	17.9	18.7	−
12	Antidiabetics, excl. Insulins	15.5	14.8	−
13	Urologicals	14.9	20.4	+
14	Anxiolytics	14.5	28.0	++
15	Selective Ca^2+^ blockers (dihydropyridines)	14.0	14.9	−
16	Antipsychotics	12.9	15.3	++
17	Low-ceiling diuretics, thiazides	11.4	13.1	−
18	MS DMDs*	11.1	15.8	−
19	Angiotensin II receptor blockers	9.9	10.3	−
20	Adrenergics, inhalants	9.5	23.1	−

DMDs: disease-modifying drugs. ATC: Anatomical Therapeutic Chemical classification system (from the WHO), shown at the third level (medication class). GERD: Gastro-esophageal reflux disease. ACE: Angiotensin-converting enzyme. Ca^2+^: Calcium. CNS: Central nervous system ‡The percentage sum exceeded 100 as individuals could fill prescriptions for more than one medication class. *ATC classification system was modified to include an “MS disease-modifying drugs” category, thus grouping all prescriptions for an MS-specific treatment available/approved in Canada during the study period (i.e. beta-interferon, glatiramer acetate, natalizumab, fingolimod, dimethyl fumarate, teriflunomide, alemtuzumab, daclizumab, and ocrelizumab). † CNS effects: ++: significant; +: minor; −/+: ambiguous; −: no effect. Attribution of each medication’s CNS effect was performed by the study’s first author (AC, trained psychiatrist and neurologist) according to the pharmacodynamic and pharmacokinetic profile of each drug class based on Brunton L, Knollmann B. Goodman and Gilman’s the Pharmacological Basis of Therapeutics, 13th Edition. McGraw-Hill Education; 2017. Medications to treat MS-related fatigue, such as stimulants and amantadine, are grouped separately, as per the third level of the ATC classification system, under “psychostimulants” and “dopaminergic agents,” respectively. We performed additional *post hoc* descriptive analyses and found that 10.6% (423/3995) of persons with polypharmacy filled a prescription for a stimulant and 3.2% (128/3995) filled a prescription for amantadine, representing 8.5% and 1.8% of the polypharmacy days, respectively.

**Figure 4.**
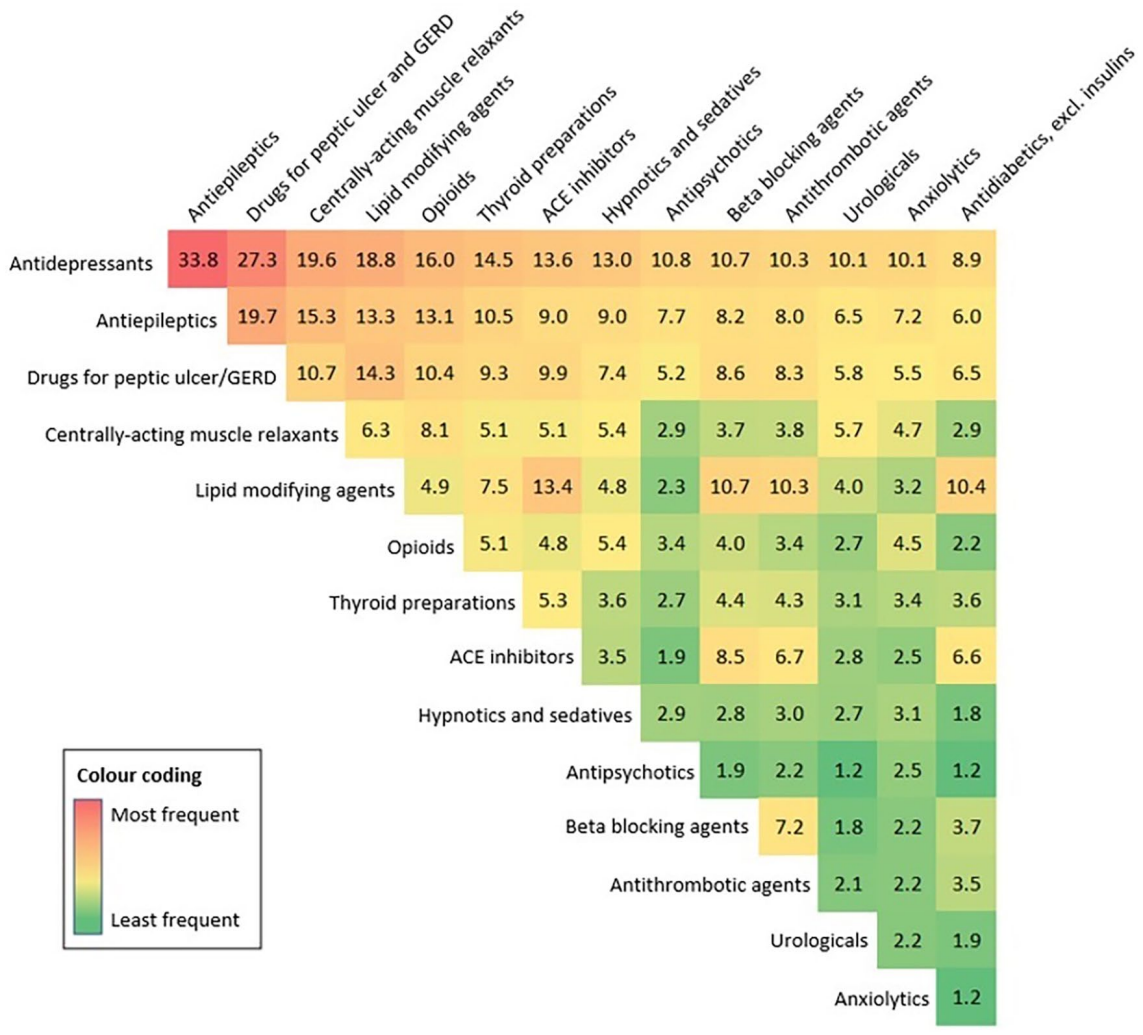
Heatmap depicting the most frequent combinations of medication classes co-prescribed in the MS population exposed to polypharmacy (reported as the percentage (%) of the total number of polypharmacy days in 2017). GERD: Gastro-esophageal reflux disease; ACE: angiotensin-converting enzyme. Note for the interested reader: a post hoc descriptive analysis for individuals filling simultaneous prescriptions for antidepressants and antiepileptics (vs. those not) was performed, focusing on additional pain-related medications used (opioids and centrally acting muscle relaxants). More than half (52.1%; 788/1513) of the antidepressant/antiepileptic co-users (vs. 38.0%; 942/2482 of the non-co-users) also filled prescriptions for opioids, representing 28.2% (112,255/398,531) and 20.0% (107,141/536,833) of their polypharmacy days, respectively. In addition, 41.2% (624/1513) of antidepressant/antiepileptic co-users (vs. 29.4%; 730/2482 of the non-co-users) filled a prescription for a centrally acting muscle relaxant, representing 31.2% (124,191/398,531) and 24.3% (130,686/536,833) of their polypharmacy days, respectively.

Antidepressants were most commonly co-dispensed with antiepileptics (33.8% of polypharmacy days included both medication classes), drugs for peptic ulcer/GERD (27.3%), centrally acting muscle relaxants (19.6%), lipid-modifying agents (18.8%), and opioids (16.0%). Other frequent combinations included antiepileptics with either drugs for peptic ulcer/GERD (19.7%) or centrally acting muscle relaxants (15.3%) (Figure 4).

### Complementary analyses

In 2017 alone, 16.0% (*n* = 2275/14,227) of individuals filled ⩾1 DMD prescription (15.8% (631/3995) of polypharmacy-exposed persons and 16.1% (1644/10,232) of those unexposed). The prevalence of polypharmacy was 27.7% (*n* = 631/2275) for these “DMD users” and 28.1% (n = 3364/11,952) for non-users. This was despite DMD users/non-users differing by age (mean ages = 45.2/57.4 years) and comorbidity (e.g. 19.2% (*n* = 436/2275) 31.2% (*n* = 3724/11,952) had ⩾1 comorbidity at the study entry). In a *post hoc* descriptive exploration, when DMD prescriptions were removed from the medication count contributing to the polypharmacy definition, polypharmacy prevalence among DMD users decreased to 20.4% (*n* = 463/2275).

Finally, the odds of any hospitalization in 2017 were 2.4 times higher for polypharmacy-exposed individuals compared to unexposed (aOR = 2.40; 95% CI: 2.20–2.63) after adjusting for sex, age, SES quintiles, and comorbidity burden.

## Discussion

In this large population-based study comprising 14,227 individuals with MS, more than one in four experienced polypharmacy during the year 2017. Furthermore, nearly two-thirds of those with polypharmacy were exposed for more than 180 days. The odds of polypharmacy were higher for women, older individuals, those with more comorbidities, and with a lower (more deprived) SES, based on neighborhood-level income. Antidepressants, antiepileptics, and drugs for peptic ulcer/GERD most frequently contributed to polypharmacy, with each representing more than 40% of the polypharmacy days. Finally, hyper-polypharmacy (⩾10 concurrent medications for longer than 30 days) affected 1 in 20 of the MS population. This extreme form of polypharmacy has been associated with harm, including a higher risk of adverse drug-related hospitalizations,^20^ and possibly higher mortality.^19^ Taken together, our findings suggest that polypharmacy is common in the MS population. While use of multiple medications may be appropriate for some individuals, the potential for unintended, negative health outcomes is worthy of further consideration.

Relatively few studies have explored polypharmacy in the MS population. This research gap was highlighted by authors of a qualitative systematic review who found only seven such studies (published between 2014 and 2019),^21^ totaling, to the best of our knowledge, nine studies to date.^11,22^ Polypharmacy, typically defined as the use of five or more medications, has been estimated to affect between 15% and 59% of persons with MS. The lowest estimate (15%) included 2276 respondents of a web-based survey who self-identified as having MS, and the highest (59%) comprised 342 patients with MS attending an MS rehabilitation center who were taking either an antiepileptic drug or a tricyclic antidepressant.^23,24^ However, in none of the MS studies exploring polypharmacy was a given period of time (e.g. >30 days) included in the definition used for assessing polypharmacy, thus creating challenges when comparing across studies. Furthermore, the majority (seven out of nine) of the study authors sourced participants from single centers, narrowing the scope and generalizability of findings. While a large, multi-site, population-based study reported that, in the year prior to study entry (1996–2014), 32%–42% of individuals with MS using a DMD filled prescriptions for ⩾5 non-MS medication classes, the study was not designed to specially examine polypharmacy.^25^ Thus, these prescriptions did not necessarily require to be filled concurrently, naturally resulting in higher polypharmacy estimates in comparison with our findings.

Polypharmacy prevalence rates increase with age and number of comorbidities, both in the general and MS population.^1,21^ We found that the results from our study, after adjusting for other characteristics, broadly concurred with these findings. In addition, we found that the odds of polypharmacy were higher for women, which was consistent with a nation-wide study from Sweden, comprising over nine million residents,^26^ although not with a single-center study of 306 individuals with MS, where no sex differences were observed.^27^ Polypharmacy maybe more common in women than men as life expectancy (and potential to accrue comorbidity) and healthcare system access is typically higher, thus increasing opportunity to receive medications.^26^ The relationship between medication use and SES has also differed across studies and settings. Although we found that the odds of polypharmacy were higher as the neighborhood-level SES decreased, a Swedish study conducted in the elderly population, also within a universal healthcare setting, found no clear association between occupation or income and polypharmacy.^28^ These differences may reflect the different study populations as well as challenges in defining and measuring SES. SES is a complex construct and can represent a diversity of factors, from location of residency (accounting for the broader wealth of the community in which a person resides) through to an individual’s education level and occupation.^29^ Nevertheless, persons living in more deprived versus affluent areas are more likely to accrue chronic diseases, which may increase polypharmacy prevalence.^29^ We were not able to find another MS study exploring the relationship between SES and polypharmacy.

The medication classes most frequently contributing to polypharmacy in our cohort were the antidepressants, followed by the antiepileptics, and the drugs for peptic ulcer/GERD. While we were unable to assess clinical appropriateness, the reason(s) behind these most commonly filled prescriptions could be for the treatment of comorbidities (for instance, depression, hyperlipidemia, or hypertension are common in MS), or other frequent MS symptoms,^4^ such as bladder dysfunction, paraesthesia, or pain. The antiepileptics gabapentin, clonazepam, and pregabalin were also frequently used in our population and are also those commonly prescribed to manage MS symptoms, including pain or spasticity.^4^ Treatment of pain may also contribute to the higher use of opioids between polypharmacy-exposed/unexposed individuals. Nonetheless, certain medications that often contribute to polypharmacy are also considered to be frequently prescribed for poorly defined reasons,^30^ and can be associated with negative health outcomes.^31^ For example, in the general population, an estimated 25%–70% of prescriptions for a proton–pump inhibitor did not have an appropriate clinical indication, and ongoing use of such medications may carry harm.^30^ Thus, our observation of frequent use of drugs for peptic ulcer/GERD in the MS population warrants further examination. We also found that the hypnotics and anxiolytics were used by over one in four polypharmacy-exposed individuals with MS in our study, representing 14%–18% of the total polypharmacy days. These medications, widely used to treat anxiety, insomnia, as well as certain MS symptoms including spasticity and tremor, can lead to chronic misuse, and have been associated with certain health risks, such as drug dependence and cognitive impairment in the general population.^31^

Polypharmacy has been associated with negative outcomes in the MS population. For example, polypharmacy (use of ⩾5 concurrent drugs) was associated with increased fatigue, subjective cognitive impairments, and poorer performance on memory tests in a cohort of 85 MS participants using a DMD (28 of whom were exposed to polypharmacy, based on self-reported medication use).^9^ In addition, the use of multiple medications and, specifically, CNS-active medications has been linked to increased risk of self-reported falls and injurious falls in persons with MS (based on diary entries made by 248 ambulatory participants).^32^ Our results show that among MS individuals with polypharmacy, co-prescription of different CNS-active medications was common. The frequent combined use of antidepressants and antiepileptics, muscle relaxants, or opioids suggest that the management of mood disorders, pain, and spasticity might represent relevant contributing factors to polypharmacy among MS individuals. However, some of these combinations may be potentially harmful. For example, opioids were combined with other CNS depressants, such as the centrally acting muscle relaxants (8.1% of the polypharmacy days), the hypnotics and sedatives (5.4%) and the anxiolytics (4.5%). These combinations have been reported by the U.S. Food and Drug Administration^33^ to be associated with increased risk of respiratory depression and death. In addition, women may be more vulnerable than men to drug-related harm.^34^ This is particularly relevant in MS, which disproportionately affects women, who were, as shown in our study, at greater risk of polypharmacy than men. Finally, our results provide preliminary insights into the link between polypharmacy and health outcomes as the odds of all-cause hospitalization (in 2017) were more than double for polypharmacy-exposed (vs. unexposed) MS individuals, adjusting for sex, age, SES, and comorbidity burden.

Our study has certain limitations. First, the actual exposure to polypharmacy may have been over- or under-estimated. On one hand, we were not able to ascertain whether the medications were taken by the individuals regularly or as prescribed. Conversely, medications not requiring a prescription or purchased over-the-counter were not captured in our dataset. The administrative data also lacked clinical information such as MS disease course or severity, or the drug indication, preventing an assessment of “polypharmacy appropriateness.” Nonetheless, the relatively high prevalence of polypharmacy found in our study suggests that exploring polypharmacy in the MS population is worthy of further consideration. We used an overall measure of comorbidity burden in our population; future work could include evaluation of individual comorbidities. In addition, while we described the occurrence of polypharmacy among DMD users, future studies may be necessary to examine more closely the relationship between the DMDs and polypharmacy. The DMD exposure rates in our population were comparable to other population-based studies conducted within universal healthcare settings (discussed elsewhere)^15^ and were also not unexpected given the short study duration and the age composition of the included cohort (mean age: 55.4 years). Strengths of our study included the use of prospectively collected administrative health data within a universal healthcare setting that captured all prescriptions filled at outpatient or community pharmacies in the province, minimizing recall bias. Furthermore, we used a validated algorithm applied to the entire population of the province of BC to identify MS cases,^15^ minimizing selection bias.

## Conclusion

Our study provides an insight into the prevalence and characteristics of polypharmacy in a “real-world” MS population. Individuals with MS were frequently exposed to polypharmacy, with women, older persons, those with a higher comorbidity burden and lower SES at higher risk. Further work is needed to better characterize and identify this risk of polypharmacy, for example, by considering participants’ specific comorbidities, or by accessing different measures of SES, such as personal or family income or level of education and employment status. In addition, assessing the impact of polypharmacy on health outcomes, such as risk of hospitalization, morbidity, and health-related quality of life, is warranted. Finally, patients and prescribers may benefit from treatment recommendations that not only address when to start a drug, but also provide guidance on a shared decision-making approach to stopping medication, as appropriate.^35^

## Supplemental Material

sj-docx-1-msj-10.1177_13524585221122207 – Supplemental material for Polypharmacy and multiple sclerosis: A population-based study Supplemental material, sj-docx-1-msj-10.1177_13524585221122207 for Polypharmacy and multiple sclerosis: A population-based study by Anibal Chertcoff, Huah Shin Ng, Feng Zhu, Yinshan Zhao and Helen Tremlett in Multiple Sclerosis Journal
