# Supplementary material for: Polypharmacy and multiple sclerosis: A population-based study
Source: Mult Scler. 2022 Oct 27;29(1):107–18. doi: 10.1177/13524585221122207 (PMC9896267; doi:10.1177/13524585221122207)
Supplement: sj-docx-1-msj-10.1177_13524585221122207 – Supplemental material for Polypharmacy and multiple sclerosis: A population-based study [file sj-docx-1-msj-10.1177_13524585221122207.docx]

**Supplementary Table. Diagnostic and drug-related codes used to identify people with multiple sclerosis using the hospital, physician and pharmacy data.**^15^

|  | **ICD-9** | **ICD-10** | **Drug identification number** |
| --- | --- | --- | --- |
| **Multiple sclerosis codes** |  |  |  |
| Multiple sclerosis | 340 | G35 | - |
| **MS-specific disease modifying drugs** |  |  |  |
| Interferon beta-1b | - | - | 02169649, 02337819 |
| Interferon beta-1a | - | - | 02237770, 02269201, 02281708, 02277492, 02237317, 02237319, 02237320, 02318253, 02318261 |
| Glatiramer acetate | - | - | 02233014, 02245619 |
| Natalizumab | - | - | 02286386 |
| Fingolimod | - | - | 02365480 |
| Dimethyl fumarate | - | - | 02404508 |
| Teriflunomide | - | - | 02416328 |
| Alemtuzumab | - | - | 02418320 |
| Daclizumab | - | - | 02459620, 02459639 |
| Ocrelizumab | - | - | 02467224 |
| Cladribine | - | - | 02470179 |

ICD: International Classification of Diseases; As per the data available, only medications accessible in Canada through until December 2017 were included.
